# Sibling Competition & Growth Tradeoffs. Biological vs. Statistical Significance

**DOI:** 10.1371/journal.pone.0150126

**Published:** 2016-03-03

**Authors:** Karen L. Kramer, Amanda Veile, Erik Otárola-Castillo

**Affiliations:** 1 Department of Anthropology, University of Utah, Salt Lake City, Utah 84112, United States of America; 2 Department of Anthropology, Purdue University, West Lafayette, Indiana, United States of America; London School of Hygiene and Tropical Medicine, UNITED KINGDOM

## Abstract

Early childhood growth has many downstream effects on future health and reproduction and is an important measure of offspring quality. While a tradeoff between family size and child growth outcomes is theoretically predicted in high-fertility societies, empirical evidence is mixed. This is often attributed to phenotypic variation in parental condition. However, inconsistent study results may also arise because family size confounds the potentially differential effects that older and younger siblings can have on young children’s growth. Additionally, inconsistent results might reflect that the biological significance associated with different growth trajectories is poorly understood. This paper addresses these concerns by tracking children’s monthly gains in height and weight from weaning to age five in a high fertility Maya community. We predict that: 1) as an aggregate measure family size will not have a major impact on child growth during the post weaning period; 2) competition from young siblings will negatively impact child growth during the post weaning period; 3) however because of their economic value, older siblings will have a negligible effect on young children’s growth. Accounting for parental condition, we use linear mixed models to evaluate the effects that family size, younger and older siblings have on children’s growth. Congruent with our expectations, it is younger siblings who have the most detrimental effect on children’s growth. While we find statistical evidence of a quantity/quality tradeoff effect, the biological significance of these results is negligible in early childhood. Our findings help to resolve why quantity/quality studies have had inconsistent results by showing that sibling competition varies with sibling age composition, not just family size, and that biological significance is distinct from statistical significance.

## Introduction

The effects of family size on offspring quality, often measured as offspring survival, growth, health or reproductive outcomes, have been the focus of many human life history studies [1, 2]. Because higher fertility can lower the availability of time, food and resources per offspring, a negative relationship between the number and quality of offspring is theoretically predicted [3–6]. Results, however, have been mixed. Negative associations between family size and child quality have been demonstrated in a number of contemporary and historic populations [7–12]. Other studies demonstrate a tradeoff between the number and quality of children, but only under circumstances where resources are limited [13]. Still other studies find either a positive relationship [14–16], or no relationship [1, 17–22] between fertility and child quality.

Empirical results may be inconsistent for several reasons. First, intra-population phenotypic variation in parental condition can pose conceptual and methodological challenges that obscure quantity/quality tradeoffs [15, 23]. For example, highly fit parents can produce large numbers of offspring that are also of high quality. This is particularly problematic with large-scale national or regional-level data where within-population phenotypic variation is substantial [9]. Second, tradeoffs may be mediated by differences in sibling competition across children’s developmental stages [24]. In high-fertility, traditional societies, younger and older siblings, or male and female children, may have different, and even opposing effects on post-weaning childhood growth [25]. Consequently, family size as an aggregate variable may obscure the source of sibling competition and confound tradeoff costs. Third, many study designs fail to distinguish biological from statistical significance [26–28]. While family size may be a statistically significant predictor of growth outcomes, the effect on growth may not be biologically meaningful in terms of health and fitness. Indeed, substantial variation exists in population growth trajectories and adult body sizes that may or may not correspond to biological fitness ([29–32]see S1 Text).

In this paper we first outline the potentially different influences that younger and older siblings may have on child quality. We then use linear mixed models to evaluate both the statistical and biological significance that family size, younger siblings and older siblings have on Maya children’s growth. We conclude by discussing the importance of disaggregating family size, and considering biological significance and population-specific growth metrics when comparing growth outcomes. Our goal is to highlight a number of methodological issues that may help resolve why family size effects on child quality have had mixed empirical support.

### Competition with younger siblings

Maternal investment is necessary for infant survival in all but the most modern of human societies. Breast milk immunity, nutrients and hormones, and intensive direct maternal care buffer nursing children from infection and nutritional disruption [33]. In most cases, maternal lactation is not substitutable and the time mothers allocate to direct infant care is relatively consistent cross culturally [34]. Mothers with both nursing and weaned children are challenged to simultaneously care for infants while also spending time in economic activities that benefit older children. In societies where maternal time allocation has been documented, mothers balance these competing demands by maintaining direct childcare but reducing time spent in either domestic activities, food production (foraging or field work) or caretaking weaned children [34–37]. While intensive maternal focus limits the infant’s exposure to sibling competition, the weanling faces a variety of new challenges.

In natural fertility populations, children typically are weaned following a subsequent maternal pregnancy or the birth of a younger sibling. Weanlings lose the protective nutritional and immunological buffer of breast milk, and maternal attention shifts from the penultimate child to the youngest child. Weanlings, who for the first time are relying only on their own immune systems, are susceptible to new health-related growth challenges [38] as maternal care is replaced or supplemented by others and they come into contact with an expanded social sphere. Increased social contact and subsequent disease exposure may help explain why stunting often occurs among recently weaned children [33, 39]. Thus, because maternal care is diverted away from weanlings, younger siblings may pose a unique threat to a young child. The effects of young siblings should be particularly pronounced when fertility is high, birth intervals are short and a child has been recently displaced by a newborn.

### Competition with older siblings

Although it is clear why younger siblings may be a competitive hazard to a young growing child, several opposing influences affect whether older siblings are a disadvantage or advantage. Older siblings may present a negative influence if disease exposure and morbidity rates correlate with family size [40, 41]. If severe enough, high communicable disease loads can compromise growth [42]. Epidemiological risks of growing up in a large family, however, likely attenuate with age as a child’s immune system matures. Older siblings also may have a negative impact in societies where wealth is generated through land acquisition, herd size, wages, or other divisible forms of income. Under these circumstances, a larger family may dilute resources available per child and older siblings may be a source of competition.

In other cases, however, older siblings may be an advantage. In many traditional foraging and agricultural societies, wealth is mediated by the size of the household labor force, which directly impacts the resources that it can produce [43]. In societies where older siblings contribute to household production and have a positive economic value, they may add to the resources available to young children. This is supported by the association between children’s economic help, higher maternal fertility and improved sibling outcomes in a number of traditional societies [43–48].

### Test predictions

We take advantage of a large, longitudinal anthropometric dataset that tracks height and weight measurements taken monthly from weaning to age five in a population of Maya subsistence agriculturalists. We focus on post-weaning growth performance because 1) it is an important proxy measure of biological fitness in traditional populations, 2) it is a life stage when children are particularly vulnerable to sibling competition, and 3) early-life growth deficits can have long-term health consequences [49]. 4) Lastly, focusing on early childhood in a population with large families allows us to simultaneously evaluate the competitive effects of younger and older siblings.

Because our test population is in the early stages of market integration and the subsistence base is still largely agricultural, parents make relatively few cash investments in children [43]. In this context, we test three predictions. 1) While a tradeoff may be evident in very large families, we expect that family size per se will not have a significant biological impact on young children’s growth. 2) Because mothers focus time and energy on infant care, and weanlings are inherently vulnerable, we expect that the number of younger siblings will have a negative effect on growth outcomes. 3) Although Maya children were traditionally productive economic contributors, because they currently spend more hours in school, we expect older siblings to neither strongly add to nor detract from resources or time available to young children, and have a neutral or negligible effect on children’s growth.

## Methods

### The study population

The Maya study community (*n* = 494) of subsistence maize agriculturalists is located in a remote area of rural Campeche, Mexico [43]. Families make their living by small-scale farming, and most food consumed is grown, although small amounts of cash may be generated through maize and honey sales. Participation in wage labor is limited, and never by children, because of distances needed to travel and the perceived low returns compared to agriculture.

Prolonged and intensive breastfeeding is the norm despite recent changes in health care access [50]. Supplemental foods are introduced at six months and children are fully weaned by two and a half (median age = 2.58, *n* = 74, 95% *CI* = 2.46–2.69). As soon as children are able to walk they are given great latitude to independently explore their environment. By age three or four, children run errands, perform simple domestic tasks and take care of their younger siblings. Compared to World Health Organization (WHO) standards, Maya children are short for their age, but are well-nourished and healthy (S1 and S2 Figs; S1 Text for discussion). Schools have been built in recent years, and most children ages six to twelve spend several hours a day attending classes, with considerable recidivism at older ages.

The Maya study case is an ideal opportunity to evaluate sibling competition for several reasons. 1) They are a high fertility population, and variation in numbers of older and younger siblings is sufficient to evaluate the effects of competition. 2) Individual-level data on parental anthropometrics and socioeconomic condition allow us to account for common measures of phenotypic variation. 3) Longitudinal monthly data from weaning to age five permit us to observe both short-term and long-term effects of sibling competition on child growth.

### Data collection

The height and weight of Maya children were collected at the beginning of each month as part of a national child health surveillance program. Measurements were conducted in a clinic by a community-based, physician-trained health promotor using government-provided standard weigh scales and stadiometers. All community mothers participate in the program with few missed monthly measurements. Children enter the program at birth and census out on their fifth birthday. Seventy-five children ages 0 to 5 were measured monthly from 2007, when the program was initiated, to 2011 (*n* = 1571 observations).

The children were measured an average of 20.9 times (*SD* = 9.2; Table 1). The health promoter also keeps a record of births, and most children’s ages are accurate to the day. Children’s birthdates and ages were cross-checked with annual censuses, including family size, the number of older siblings and younger siblings, collected by Kramer and maintained in a longitudinal database since 1992. Maternal heights were collected in 2010. This is a subsistence agricultural economy, and wealth status is measured as the number of hectares a family has under cultivation. During the same period that the children’s anthropometric data were collected, each plot that a family has under cultivation was measured using GPS technology to calculate total area under cultivation.

**Table 1:** Sample characteristics for Maya children ages 2.5–5.0, showing mean, standard deviation and range in parentheses. Age, height and weight are averaged across the 5-year sample. Family size, number of older siblings and number of younger siblings are averaged from each child’s last measurement.

Variable	Male	Female	Total
Sample size (individuals)	39	36	75
Number of measures/child	19.3 ± 9.6 (1–30)	22.7 ± 8.5 (5–30)	20.9 ± 9.2 (1–30)
Age	3.8 ± 0.7 (2.5–5)	3.8 ± 0.7 (2.5–5.0)	3.8 ± 0.7 (2.5–5)
Height	91.1 ± 5.6 (76–105)	88.9 ± 5.6 (75–106)	90.0 ± 5.7 (75–106)
Weight	14.1 ± 1.9 (9.4–19.5)	13.2 ± 1.6 (9.4–18.8)	13.6 ± 1.8 (9.4–19.5)
Family size	3.7 ± 1.7 (1–8)	4.3 ± 2.4 (1–9)	4.0 ± 2.1 (1–9)
Older Siblings	1.8 ± 1.9 (0–7)	2.6 ± 2.5 (0–8)	2.2 ± 2.2 0–8)
# of younger siblings	0.82 ± 0.9 (0–3)	0.70 ± 0.7 (0–2)	0.76 ± 0.84 (0–3)

Written permits for research were secured from the local government and health promoter. Consent on behalf of the children was obtained verbally from mothers (or fathers if mothers were unavailable) during household visits. Written consent was waived because many parents are illiterate and the research presented no more than minimal risk of harm to subjects and involved no procedures for which written consent is normally required outside of the research context. These research protocols and consent procedures were approved by Harvard University’s Institutional Review Board and the University of Utah’s Institutional Review Board.

#### Sample considerations

Three children from one very large family (14 children total, 10 children living at home) were excluded from the analyses as an isolated case. Although the young children were growing adequately (mean WAZ = 0.89, mean HAZ = 0.71 based on measurements taken at age 3), the family’s size is not representative. When retained in the models, the strength of the interactions between child size and age substantially increases; removing them as outliers dampens the interaction effect.

In the final analysis sample, family size, measured as number of offspring aged 15 and younger living in the household including the child being measured, ranged from 1–9. We further narrow the sample to observations of children between the age 2.5 (median observed age at weaning) and 5.0 because breastfed infants tend to be buffered from the effects of sibling competition, and variation in growth during the first two years of life is often a response to birth weight rather than to exogenous factors [51, 52]. The 2.5–5.0 age range allows us to focus on family composition without these confounding influences.

### Model construction

We test our predictions by constructing a series of linear mixed models (LMM) fit by REML (NLME package [53] in the R computing environment) [54]. Models were constructed for the outcome variables *height* and *weight*. One set of models was constructed for the predictor variable *family size* (the number of children 15 and younger living in the household, including the child being measured). Because our primary interest is in the differential effects of family composition, and because family size and older siblings are highly correlated, a second set of models was constructed with predictors that disaggregate family size. We included both the *number of younger siblings* and the *number of older siblings* in the same model, and hold one constant to evaluate their respective effects separately. The value of the predictor variables is assessed each time a child is measured. *Family size*, *younger siblings* and *older siblings* therefore change as older siblings leave and new children are born into the family.

We accounted for the non-independent error across several factors by treating these models as a nested repeated-measure LMM. Children were measured multiple times, therefore *child ID* was treated as a random effect. In addition, many children have the same mother and their growth responses will be correlated and not independent. To account for this, *child’s id* was nested within *mother’s id* and treated as a random effect.

The full model for the predictor *family size* and the outcome variables *height* and *weight* includes the random effects *child’s id* nested within *mother’s id*, the control variables *age* (recorded at each measurement time point) and *sex*, and the covariates *maternal height* and family *wealth status* to account for phenotypic variation (S2 Text). Three interaction terms—age*sex, age*predictor, and sex*predictor—were also added. The full model for the predictor variables *younger siblings* and *older siblings* and the outcome variables *height* and *weight* includes the same random effects and control variables as above, and sex and age interaction terms for both predictors. Best-fit models for each predictor variable were selected by comparing AIC values when variables were dropped from the full model using backward selection. If the change in AIC between two models differed by ≤2, the model with fewer predictor variables was selected.

### WHO-defined stunting and the use of population-specific Z-Scores

The WHO defines stunting as children’s whose height is <-2 HAZ-scores below the mean for a sample of global populations. This guideline is used to identify children who are potentially at risk of failing to thrive. In several populations children below this threshold also have been linked to negative health and fitness outcomes [55–60], including greater childhood morbidity and mortality [56, 59]. Short adult stature is also associated with reduced reproductive success; shorter women have smaller babies and more obstetric complications [61–63], whereas shorter men have fewer reproductive partners [64, 65]. The detrimental downstream effects of stunting are so pronounced that in 2012 the WHO adopted a resolution to reduce the number of stunted under-five children by 40% by 2025 [66].

Mean WHO HAZ-scores for the 75 Maya boys and girls in the sample are -2.95 and -2.72, respectively (S1 Text). Adult height for 20–40 year olds averages 143.2 cm for females and 156.1 cm for males. Although Maya children and adults would be classified as stunted by WHO criteria, this does not meaningfully reflect Maya health or fitness for a number of reasons. Children and adults are well nourished; BMIs for all children ages 10–20 are within the 50^th^ to 90^th^ percentiles, depending on age and sex (average male BMI for 10–20 year olds = 20.4, *SD* = 3.0, *n* = 61; average female BMI = 21.9, *SD* = 3.5, *n* = 51). Surviving fertility for women 40 and older is 6.4 (*SD* = 2.8, *n* = 60), and has remained unchanged over the last 20 years (*t* = 1.38, *p* = .1741, *n* = 52). Ninety-eight percent of children born survive to age 16 [43, 67] (S1 Text). Birthweights documented since 2002 are within the WHO range of normal (mean 3.04 kg, *SD* = 0.49, *n* = 109). Of this sample, 9% are low birth weight (LBW) babies, the same percent of LBW reported for Mexico [68]. Although Maya stature might increase with different dietary inputs and life styles [69], these characteristics strongly suggest that while short by WHO standards, their stature is not critically compromising to their health and fitness. Consequently, we use Maya population-specific Z-scores as a more biologically relevant metric of within-population comparisons of children’s growth in families of varied composition [70] (see S1 Text for calculation of population-specific Z-scores). We display WHO Z-scores (S1 Fig, S2a and S2b Table), but only for the purpose of situating the Maya within a cross-cultural perspective.

### Biological significance criteria

We emphasize that the magnitude of a parameter estimate needs to have a biological, not just a statistical impact on growth. We differentiate between these measures of significance because there is tremendous variation in population growth trajectories and size, not all of which may have biologically meaningful fitness impacts [32]. We expect that if a parameter estimate is statistically significant, but very small, it may be of little consequence to early childhood health or fitness. We define biological significance using the following two criteria. 1) If the Maya population-specific Z-score is <-2 for any given number of siblings, we consider the sibling effect to be biologically significant. 2) If the relative change in Maya population-specific Z-scores is associated with a >2 decrease as sibling number increases, we consider this to be biologically significant.

We retain the WHO threshold of -<2 Z-scores but apply it to Maya-specific body size distributions. We do this because children below the -2 Z-score threshold represent a substantial deviation from mean body size within their population (the smallest 2.5%). We expect that individuals falling below this threshold would be at increased health risks and longer-term fitness compromises. Although a population-specific threshold for stunting is not often employed in the growth literature (but see [71–74]), the WHO standard is not appropriate to the Maya. Under such circumstances it is common practice for authors to assign a reasonable effect size in the absence of established biological significance criteria [75]. Finally, although our criteria do not directly assess long-term fitness outcomes in Maya children, body size and growth are common proxies of fitness used in life history analyses [4, 23, 76].

To establish biological significance, we use parameter estimates from the best-fit models (S1 Table) to calculate the predicted height (cm) and weight (kg) of Maya children at age 2.5 and 5.0. For the first criteria we computed the difference in predicted height and weight with each additional increase in family size, older or younger sibling. Predicted height and weight values were then converted into Maya population-specific Z-scores (see S1 Text). For the second criteria we calculate the relative change in Maya population-specific Z-scores with each increase in family size or addition in the number of younger and older siblings.

## Results

### Family size effects on young children’s growth

**Fig 1:**
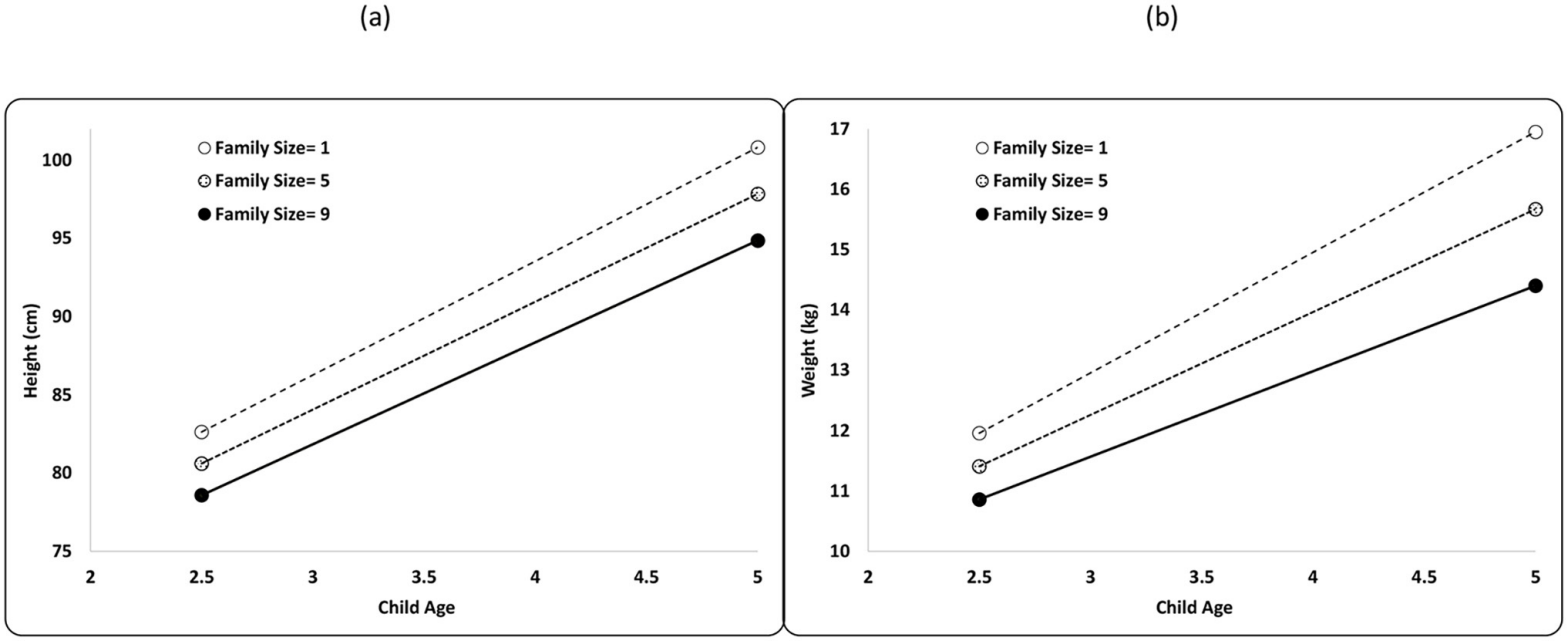
Growth performance stratified by family size (FS) for Maya children ages 2.5–5.0. Lines plot predicted (A) height (cm) and (B) weight (kg) by age for boys and girls in a family with 1, 5 and 9 children (drawn from S1 Table Models 1a-b).

The addition of a family member is slightly negatively associated with child height and weight at 2.5 years of age, and the negative effects of additional family members on growth increase with age (Fig 1, S1 Table Models 1a and 1b). The best-fit model’s parameter estimates predict that the height and weight of a 2.5-year-old Maya child decreases by 0.5 cm and 0.14 kg (or -0.17 HAZ and -0.11 WAZ), respectively, for each increase in family size (Fig 1, Table 2). The predicted height and weight of a 5-year-old Maya child decreases by 0.7 cm and 0.32 kg, (or -0.19 HAZ and -0.19 WAZ), respectively, for each increase in family size (Fig 1, Table 2, S2a and S2b Table).

**Table 2:** Percent and absolute change in height (cm) and weight (kg) with each addition in family composition. Values taken from best-fit model results in S2a and S2b Table. Boys and girls are reported separately for younger siblings and height because the interaction with gender is significant in this case (S1 Table).

	Height		Weight
**Family Size**	Boys & Girls		Boys & Girls
Age 2.5 Age 5.0	-0.6% (-0.5 cm)-0.7% (-0.7 cm)		-1.2% (-0.14 kg)-1.9% (-0.32 kg)
**Younger Siblings**	Boys	Girls	
Age 2.5 Age 5.0	1.5% (1.3 cm)-0.2% (-0.2 cm)	0.8% (0.7 cm)-0.8% (-0.8cm)	0.5% (.06 kg)-3.0% (-0.5 kg)
**Older Siblings**			
Age 2.5 Age 5.0	-0.4% (-0.3 cm)-0.6% (-0.6 cm)		0.25% (.03 kg)-0.9% (-.15 kg)

### Younger sibling effects on young children’s growth

**Fig 2:**
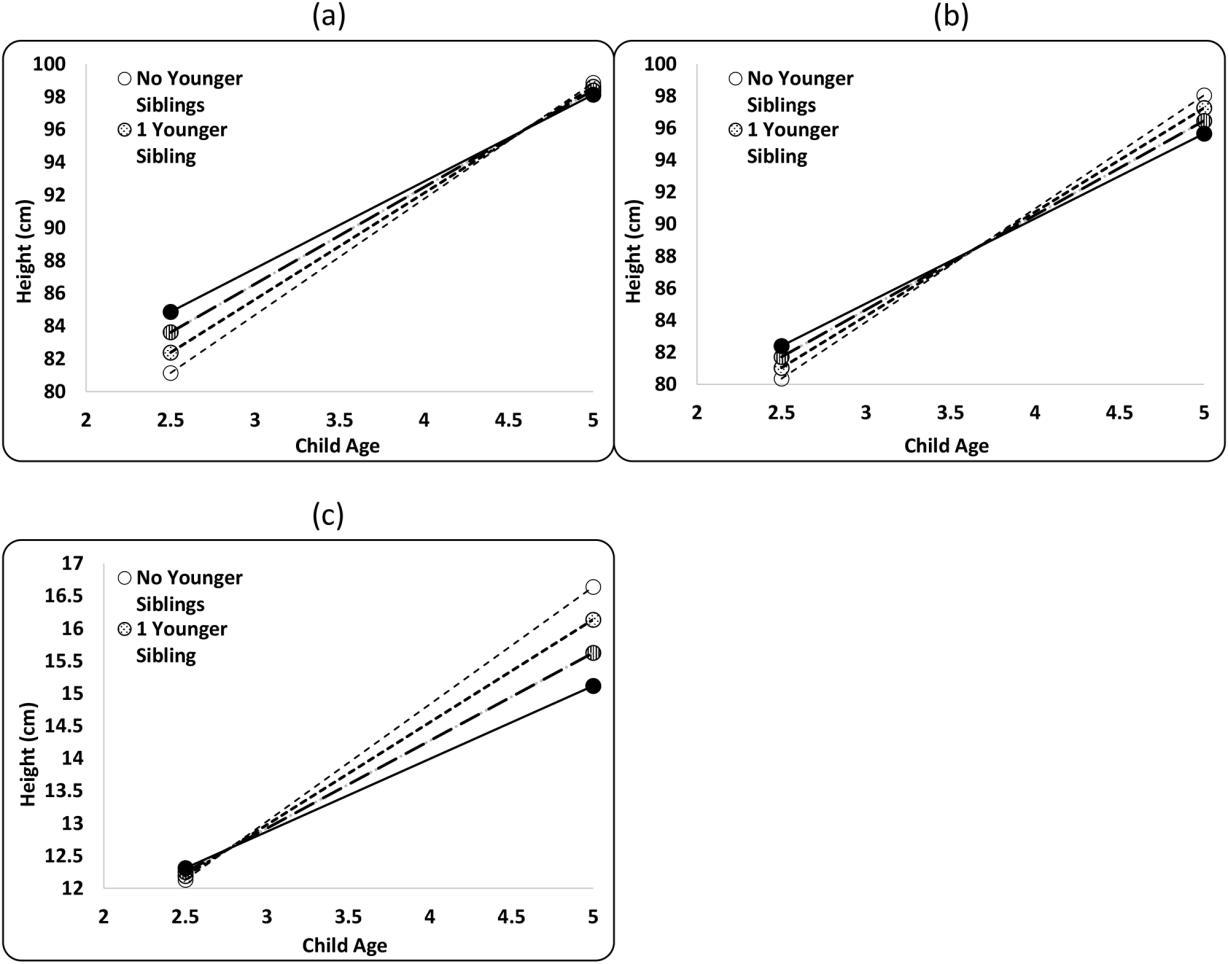
Growth performance stratified by the number of younger siblings (YS) for Maya children ages 2.5–5.0. Lines plot predicted height (cm) in (A) boys and (B) girls and predicted weight (kg) for (C) girls and boyswith 0, 1, 2, and 3 younger siblings (drawn from S1 Table Models 2a-d).

The addition of a younger sibling (holding older siblings constant) is positively associated with child height at 2.5, but negatively associated with child height by age 5 (Fig 2, S2a Table). The best-fit model’s parameter estimates predict that at 2.5, the height of a Maya boy and girl increases by 1.3 cm and 0.7 cm (or 0.57 HAZ and 0.21 HAZ), respectively, per younger sibling. Younger siblings become negatively associated with height by age 5, and the predicted height of a Maya boy and girl decreases by 0.2 cm and 0.8 cm (or -0.06 HAZ and -0.22 HAZ), respectively, per younger sibling (Fig 2, Table 2, S1 Table Model 2a).

The addition of a younger sibling (holding older siblings constant) is positively associated with child weight at age 2.5, but negatively associated with weight by age 5 (Fig 2c, S1 Table Model 2b). Though girls are lighter than boys, the magnitude of the interaction does not differ between them. The best-fit model’s parameter estimates predict that at age 2.5, the weight of a Maya child increases by 0.06 kg (0.05 WAZ) for additional younger sibling, while by age 5, their weight decreases by 0.5 kg (-0.33 WAZ) with each additional younger sibling (Fig 2c, Table 2). This is the greatest per-sibling effect we find on child growth, and accounts for a large portion of the decrease in weight observed for family size (-1.9% per family member).

### Older sibling effects on young children’s growth

**Fig 3:**
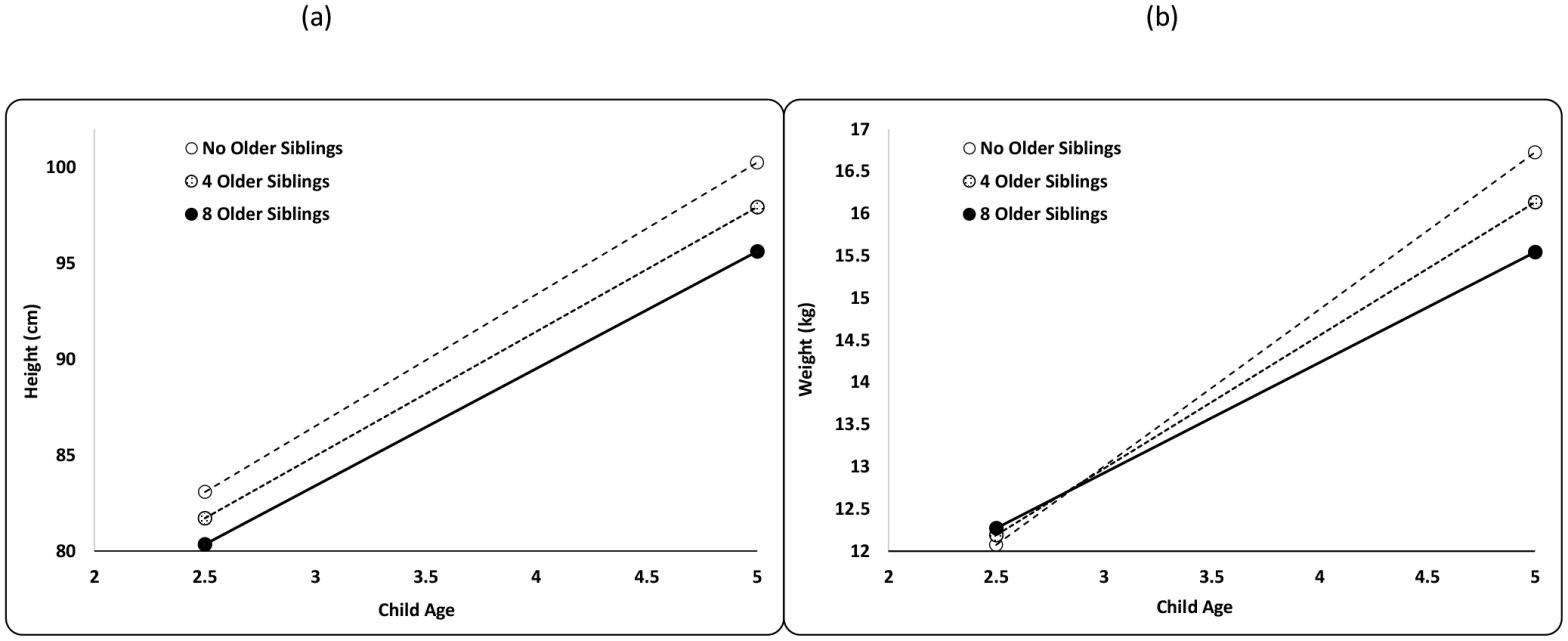
Growth performance stratified by the number of older siblings (OS) for Maya children ages 2.5–5.0. Lines plot (A) predicted height (cm) for boys and girls, and predicted weight (kg) for (B) boys and girls with 0, 4 and 8 older siblings (drawn from S1 Table Models 3a-c).

The addition of an older sibling (holding younger siblings constant) is negatively associated with child height at 2.5 years of age and the negative effect increases with age (Fig 3, S1 Table Model 2a). Height of a 2.5-year-old Maya child decreases by 0.3 cm (-0.13 HAZ), and the height of a 5-year old decreases by 0.6 cm (-0.14 HAZ) with each additional older sibling (Fig 3, Table 2).

The addition an older sibling (holding the number of younger siblings constant) is positively associated with child weight at age 2.5, but starting at 3.0 years of age, younger siblings are negatively associated with weight (Fig 3, S1 Table Model 2b). Although girls were lighter than boys, the magnitude of the interaction did not differ between them. The best-fit model’s parameter estimates predict that at 2.5 years of age, the weight of a Maya child increases by 0.03 kg (0.02 WAZ) for additional younger sibling, while by 5 years of age, weight of a Maya child decreases by 0.15 kg *(*-0.09 WAZ) for each additional younger sibling (Fig 3, Table 2).

### Biological significance

As per our criteria, detrimental effects of sibling composition on children’s growth is considered biologically significant if: 1) the Maya population-specific Z-score is <-2; or 2) the relative change in Maya population-specific Z-scores is associated with a >2 decrease as sibling number increases. Based on these criteria, Maya children’s population-specific mean height and weight Z-scores do not reach thresholds of biological significance for any value of family size, nor for any number of younger or number of older siblings at age 2.5 or 5.0 (Table 3; S2a and S2b Table). According to these criteria, siblings do not have a biologically meaningful detrimental effect on young children’s growth in the Maya, even in very large families.

**Table 3:** Summary of biological significance based on two criteria: 1) Maya population-specific Z-scores < -2; 2) change in Maya population-specific Z-score >2. Relative scores are drawn from S2a and S2b Table, using the computed delta Z-score for height and weight at family sizes of 1 vs. 9, younger sibling numbers of 0 vs. 3, and older sibling numbers of 1 vs. 8.

Predictor	Age	Sex	Outcome	Maya Z <-2	Maya Z Δ>2	Biological Significance Criteria Met
*Family Size*	2.5	Both	Height	No	No	No
	5.0	Both	Height	No	No	No
	2.5	Both	Weight	No	No	No
	5.0	Both	Weight	No	No	No
*Younger Sibs*	2.5	Boys	Height	No	No	No
	5.0	Boys	Height	No	No	No
	2.5	Girls	Height	No	No	No
	5.0	Girls	Height	No	No	No
	2.5	Both	Weight	No	No	No
	5.0	Both	Weight	No	No	No
*Older sibs*	2.5	Both	Height	No	No	No
	5.0	Both	Height	No	No	No
	2.5	Both	Weight	No	No	No
	5.0	Both	Weight	No	No	No

## Discussion

The quantity/quality tradeoff predicts that siblings in large families compete for limited parental resources and consequently children’s growth is expected to be negatively associated with family size. While several studies have found a negative family size effect on growth [12, 49, 77], others have not [20–22, 78]. Using a large, longitudinal panel study and accounting for differences in parental condition, we focus our analysis on young children where the negative impacts of sibling competition are potentially most concentrated. While we find statistical evidence of a quantity/quality effect, the biological significance of these results appears minimal during early childhood, even at large family sizes. Maya children never fall below the <-2 criteria for population-specific Z-scores, nor do they lose >2 Z-scores at any number of siblings. These results raise questions about methodological approaches to sibling competition, the meaning of statistical versus biological significance in growth studies, and the use of standard references when comparing within population growth variation.

### Sibling competition

We have suggested that in high fertility societies, family size can conflate the potentially differential effects that younger and older children have on sibling competition. Nursing siblings, who monopolize much of a mother’s time, may directly compete with recently weaned children. This follows with our finding that competition with younger siblings poses the greatest per-sibling compromise to young Maya child’s growth. This is consistent with the Godoy et al. [79] study in which stunted Bolivian Tsimane children’s catch-up growth decreased with each additional younger sibling. Similar results have also been reported for the Yanomamo, the Hadza and the Ngandu [11, 80, 81]. In the Maya case, younger siblings have a more pronounced effect on growth at age 5.0 than at age 2.5, suggesting that the buffering advantages of breastfeeding persist for some time after weaning or that allocare is preferentially directed toward weanlings.

We have proposed that older siblings may have either a positive or negative effect depending on their economic value, the level of market integration and cash outlays or other divisible forms of wealth that parents invest in children. As predicted, despite growing up in large families, older Maya children negligibly affect their younger sibling’s growth performance. The Maya are in the earliest stages of market integration, and older children neither draw down nor augment household wealth. Here we parsed family size into younger and older siblings to disaggregate different kinds of pressure on parental investment and competition among siblings. In other ethnographic contexts, the potentially differential influence of siblings may be meaningful disaggregated in other ways.

### Biological vs. statistical significance

We emphasize that while family composition, or other exogenous factors, may be statistically associated with growth, the question has to answered, is it biologically meaningful? While biological significance is not addressed in most growth model results [12, 25, 77], this under-reported aspect of growth analyses is critical to determine whether family size and sibling competition actually compromise future health and reproductive outcomes.

We have used a cutoff of <-2 population-specific Z-scores and a decrease of >2 population-specific Z-scores to assess biological significance. Given these criteria, our findings suggest that, although the Maya children in our sample are small, this is not largely attributable to sibling competition in early childhood. Further, their size appears not to be fitness compromising. Both fertility and child survivorship are high compared to many traditional populations [43, 47], and birth weights, the proportion of LBW births and BMI performance in older children are all within normal ranges (see above). Although short maternal stature is associated with obstetric complications in other populations [61–63], surviving fertility, detailed reproductive histories and structured interviews with the community’s midwife and older women corroborate that rates of maternal and infant mortality (one woman died in childbirth over the last 30 years)were low even before western biomedical care was available [43].

To address whether statistical effects are accompanied by biologically meaningful effect sizes, we used a -2 Z-score threshold as a common indictor of substantial deviation from the population mean for body size (the smallest 2.5%). We note however, that for other study populations or research questions, a more or less conservative value may be appropriate. Indeed, our longitudinal Maya life history project will help to determine a potentially more appropriate population-specific effect size in the future.

### Population-specific standards

While the WHO reference standards were recently adjusted to reflect broad patterns of global child growth variation and are often used to gauge nutritional status [82], we use population-specific Z-scores for several reasons. The short stature of the Maya is not an indicator that they have limited caloric intake, are in poor health or have compromised fertility as adults. Standard references may not be sensitive to the range of healthy growth or genetic constraints in some populations [74]. We would expect that the reaction norm for healthy growth to express variation and be sensitive to ecological context. We include Z-scores derived from the WHO standards for cross-cultural comparative purposes, but emphasize that a population-specific metric is more appropriate for within group comparisons, in this case the effects of sibling competition on growth. This approach has gained traction in recent years [71–74].

Lastly, our results raise a question about why we find minimal evidence for a quantity/quality tradeoff in early childhood. Reasonably, the more offspring a mother has, the less time, food or resources are available per individual. Among the Maya, as in most societies, investment in children is not limited to parents, but is distributed across many helpers [24, 25, 46, 83–87]. Consequently the relationship between sibling competition, family composition and growth outcomes is expected to be mediated by the availability of nonmaternal help [24, 88]. We also expect it to be mediated by how wealth is produced. Wealth in Maya society was traditionally generated through household labor and the agricultural production of parents and children. Beside their economic contributions, Maya children spend substantial time providing childcare, especially to weanlings [29]. For these reasons, we expect that the economic value of children and the time they spend in childcare are important determinants of whether older siblings, and consequently family size, limit the per capita investment available for other children.

Among the Maya and other modernizing populations, traditional factors that affect the quality of offspring (children’s economic value, living in extended families, the availability of nonmaternal help and patterns of disease transmission) are in transition. Because parents can distribute their time and resources among children in novel ways (e.g., education, cash inputs), it is not surprising that quantity/quality tradeoff studies across diverse societies that differ in their level of market integration are divided in their findings. Family size may well have a negative impact on child growth under many circumstances. However, the relationship between the number and size of offspring is likely context-specific. In those societies where wealth is generated by family labor, children’s economic value is high and allocare is common, we would predict that family size and older siblings are not significant predictors of negative child outcomes. However, in societies where wealth is divisible (generated through wages, land or herd size), the economic value of children is low or non-parental sources of help are limited, we predict a quantity/quality tradeoff to be more evident, and older and younger siblings both a source of competition.

#### Limitations

This study has a limited age focus on early childhood from the post-weaning period to five years old. During this period we find minimal evidence of a biologically meaningful quantity-quality tradeoff. Because of the short duration studied, we cannot directly compare our results with studies that cover a broader range of child development stages. The negative interaction of siblings and child age suggests that the negative relationship might increase over time. In future studies, continued measurements of child growth in this population will allow us monitor the longer term effects of sibling competition.

## Conclusion

Our results show that young Maya children’s growth is not compromised in a biologically meaningful way by sibling competition and growing up in large families. The quantity/quality tradeoff is complex in humans because cooperation and the exchange of resources and childcare necessary for growth and survival extend well beyond what parents provide. Family size and sibling competition rather than having a universally negative effect on child quality, are likely context-specific. Our findings suggest that 1) siblings of different ages can have different effects on children’s growth, and family size as an aggregate variable may not capture this distinction or the source of sibling competition. 2) Statistical significance may not reflect a biologically meaningful effect size. In the case of this study and the criteria used, sibling competition was not found to have biologically significant effects on young children’s growth. 3) This lead to our final point that population-specific Z-scores for many traditional and transitioning societies may be a more appropriate metric for research questions about determinants of within-population growth performance.

## Supporting Information

S1 Fig WHO calculated Z-scores for Maya boys (*n* = 39) and girls (*n* = 36) ages 2.5–5.0 for (A) height and (B) weight. Graph plots monthly measurements taken from 2007–2011.
(TIF)

S2 Fig Population-specific Z-scores for Maya boys (*n* = 39) and girls (*n* = 36) ages 2.5–5.0 for (A) height and (B) weight. Graph plots monthly measurements taken from 2007–2011.
(TIF)

S1 Table Best-fit models for height and weight and predictor variables. See main text for explanation of how models were calculated.
(DOCX)

S2 Table **(A) Predicted estimates from best-fit models for height (cm)**. Includes population-specific Z-scores (Maya HAZ), WHO Z-scores (WHO HAZ), and Z-score changes (Maya HAZ Δ and Who HAZ Δ) at age 2.5 and age 5.0 for each predictor variable (see main text and S1 Text for explanation of calculation). For predictor *younger siblings*, boys and girls are calculated separately because of the sex*younger siblings interaction effect. **(B) Predicted estimates from best-fit models for weight (kg)**. Includes population-specific Z-scores, WHO Z-scores, and Z-score changes at age 2.5 and age 5.0 for each predictor variable (see text and S1 Text for explanation of calculation).
(DOCX)

S1 Text Constructing WHO and population-level Z-scores. (DOCX)

S2 Text Fertility, maternal age, height and wealth status. (DOCX)
