# Supplementary figures and images for: Sibling Competition & Growth Tradeoffs. Biological vs. Statistical Significance
Source: PLoS One. 2016 Mar 3;11(3):e0150126. doi: 10.1371/journal.pone.0150126 (PMC4777386; doi:10.1371/journal.pone.0150126)

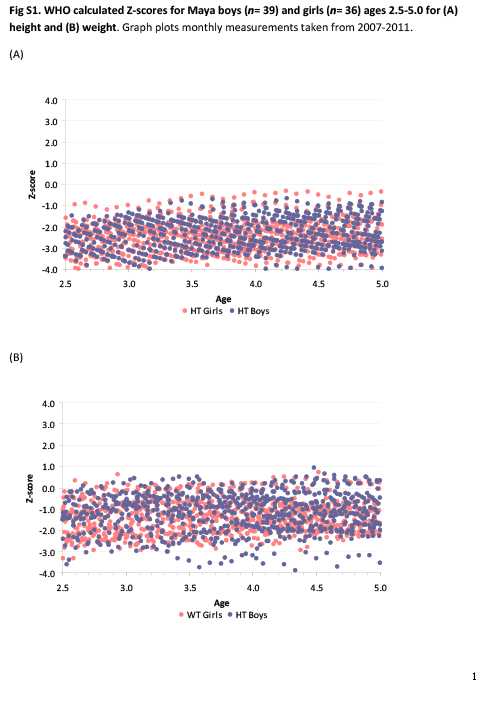

Supplement: S1 Fig — Graph plots monthly measurements taken from 2007–2011. (TIF) [file pone.0150126.s001.tif]

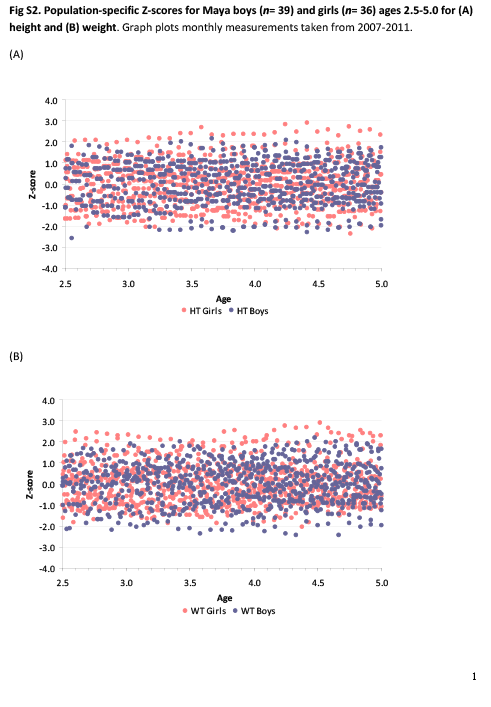

Supplement: S2 Fig — Graph plots monthly measurements taken from 2007–2011. (TIF) [file pone.0150126.s002.tif]
