# Supplementary material for: Sibling Competition & Growth Tradeoffs. Biological vs. Statistical Significance
Source: PLoS One. 2016 Mar 3;11(3):e0150126. doi: 10.1371/journal.pone.0150126 (PMC4777386; doi:10.1371/journal.pone.0150126)
Supplement: S1 Table — See main text for explanation of how models were calculated. (DOCX) [file pone.0150126.s003.docx]

**Table S1. Best-fit models for height and weight and predictor variables.** See main text for explanation of how models were calculated.

**Model 1a.** Family Size (Height) **Model 1b.** Family Size (Weight)

|  | Parameter estimate | Standard Error | T-value | p-value | Parameter  estimate | Standard Error | T-value | p-value |
| --- | --- | --- | --- | --- | --- | --- | --- | --- |
| Intercept | 44.31347 | 11.322434 | 3.91378 | 0.0001 | -0.9225071 | 5.173215 | -0.17832 | 0.8585 |
| Sex | -0.89216 | 0.677623 | -1.31660 | 0.1923 | -0.4384491 | 0.309515 | -1.41657 | 0.1611 |
| Child Age | 7.36576 | 0.084121 | 87.56195 | 0.0000 | 2.0667062 | 0.036217 | 57.06412 | 0.0000 |
| Family Size | -0.26813 | 0.117982 | -2.27260 | 0.0232 | 0.0430561 | 0.051405 | 0.83759 | 0.4024 |
| *Maternal Ht | 0.14402 | 0.076199 | 1.89006 | 0.0630 | 0.0591521 | 0.034810 | 1.69930 | 0.0938 |
| Wealth (HA) | 0.15294 | 0.172002 | 0.88915 | 0.3770 | -0.0649842 | 0.078613 | -0.82663 | 0.4113 |
| Age*Family Size | -0.09507 | 0.018173 | -5.23144 | 0.0000 | -0.0723607 | 0.007819 | -9.25443 | 0.0000 |

*Random effects: Child ID, Maternal ID Random effects: Child ID, Maternal ID*

*AIC= 4514.436, n=73, obs=1511 AIC=1983.181, n=73, obs=1511*

******Height data are missing for two mothers***

**Model 2a.** Younger and Older Siblings (Height) **Model 2b.** Younger and Older Siblings (Weight)

|  | Parameter  estimate | Standard  Error | T-value | p-value | Parameter estimate | Standard Error | T-value | p-value |
| --- | --- | --- | --- | --- | --- | --- | --- | --- |
| Intercept | 62.98339 | 1.2143966 | 51.86394 | 0.0000 | 7.180098 | 0.461672 | 15.55237 | 0.0000 |
| Maternal Age | 0.02888 | 0.0143756 | 2.00915 | 0.0447 | N/A | N/A | N/A | N/A |
| Sex | -0.79453 | 0.6608036 | -1.20238 | 0.2332 | -0.76727 | 0.283902 | -2.70258 | 0.0086 |
| Child Age | 7.46397 | 0.0792076 | 94.23301 | 0.0000 | 2.086947 | 0.033863 | 61.62936 | 0.0000 |
| Older Siblings | -0.10558 | 0.1632237 | -0.64684 | 0.5198 | 0.205285 | 0.071816 | 2.85849 | 0.0056 |
| Younger Siblings | 2.72748 | 0.4286194 | 6.36341 | 0.0000 | .627956 | 0.133427 | 4.70636 | 0.0000 |
| Age*Older Siblings | -0.09453 | 0.0179974 | -5.25241 | 0.0000 | -0.07056 | 0.007734 | -9.12377 | 0.0000 |
| Age*Younger Siblings | -0.59323 | 0.0663845 | -8.93622 | 0.0000 | -0.22725 | 0.028979 | -7.84191 | 0.0000 |
| Sex*Older Siblings | N/A | N/A | N/A | N/A | N/A | N/A | N/A | N/A |
| Sex*Younger Siblings | -0.56167 | 0.203936 | -2.75414 | 0.006 | N/A | N/A | N/A | N/A |

*Random effects: Child ID, Maternal ID Random effects: Child ID, Maternal ID*

*AIC= 4634.988, n=75, obs=1571 AIC=2034.123, n=75, obs=1571*
