# Supplementary material for: Sibling Competition & Growth Tradeoffs. Biological vs. Statistical Significance
Source: PLoS One. 2016 Mar 3;11(3):e0150126. doi: 10.1371/journal.pone.0150126 (PMC4777386; doi:10.1371/journal.pone.0150126)
Supplement: S2 Table — (A) Predicted estimates from best-fit models for height (cm). Includes population-specific Z-scores (Maya HAZ), WHO Z-scores (WHO HAZ), and Z-score changes (Maya HAZ Δ and Who HAZ Δ) at age 2.5 and age 5.0 for each predictor variable (see main text and S1 Text for explanation of calculation). For predictor younger siblings, boys and girls are calculated separately because of the sex*younger siblings interaction effect. (B) Predicted estimates from best-fit models for weight (kg). Includes population-specific Z-scores, WHO Z-scores, and Z-score changes at age 2.5 and age 5.0 for each predictor variable (see text and S1 Text for explanation of calculation). (DOCX) [file pone.0150126.s004.docx]

**Table S2a.** **Predicted estimates from best-fit models for height (cm).** Includes population-specific Z-scores (Maya HAZ), WHO Z-scores (WHO HAZ), and Z-score changes (Maya HAZ ∆ and Who HAZ ∆) at age 2.5 and age 5.0 for each predictor variable (see main text and S1 Text for explanation of calculation). For predictor *younger siblings*, boys and girls are calculated separately because of the *sex*younger siblings* interaction effect.

|  | HT  2.5 | MayaHAZ  2.5 | Maya HAZ ∆  2.5 | WHO  HAZ  2.5 | WHO HAZ ∆  2.5 | HT  5.0 | Maya HAZ  5.0 | Maya HAZ ∆  5.0 | WHO  HAZ  5.0 | WHO HAZ ∆  5.0 |
| --- | --- | --- | --- | --- | --- | --- | --- | --- | --- | --- |
| Family Size (Boys and Girls) | | | | | | | | | | |
| 1  2  3  4  5  6  7  8  9 | 82.63  82.13  81.62  81.11  80.61  80.10  79.60  79.09  78.59 | \| 0.65  0.47  0.30  0.11  -0.05  -0.23  -0.41  -0.58  -0.76 \| \| --- \| | 0.00  -0.17  -0.35  -0.52  -0.70  -0.87  -1.05  -1.23  -1.41 | -2.37  -2.50  -2.64  -2.78  -2.92  -3.06  -3.20  -3.34  -3.47 | 0.00  -0.13  -0.27  -0.41  -0.55  -0.69  -0.83  -0.97  -1.10 | 100.81  100.07  99.32  98.58  97.84  97.09  96.35  95.61  94.86 | \| 0.83  0.64  0.45  0.26  0.06  -0.13  -0.33  -0.53  -0.73 \| \| --- \| | 0.00  -0.19  -0.38  -0.57  -0.77  -0.96  -1.16  -1.36  -1.56 | -2.02  -2.19  -2.36  -2.53  -2.70  -2.87  -3.04  -3.20  -3.38 | 0.00  -0.17  -0.34  -0.51  -0.68  -0.85  -1.02  -1.18  -1.36 |
| Younger Siblings (Boys) | | | | | | | | | | |
| 0  1  2  3 | 81.14  82.39  83.63  84.88 | -0.12  0.45  1.00  1.55 | 0.00  0.57  1.12  1.67 | -2.93  -2.59  -2.25  -1.91 | 0.00  0.34  0.68  1.02 | 98.86  98.62  98.40  98.14 | 0.28  0.22  0.17  0.10 | 0.00  -0.06  -0.11  -0.18 | -2.52  -2.57  -2.62  -2.68 | 0.00  -0.05  -0.1  -0.16 |
| Younger Siblings (Girls) | | | | | | | | | | |
| 0  1  2  3 | 80.35  81.03  81.71  82.40 | 0.04  0.25  0.45  0.66 | 0.00  0.21  0.41  0.62 | -2.84  -2.65  -2.47  -2.28 | 0.00  0.19  0.37  0.56 | 98.06  97.26  96.46  95.66 | 0.17  -0.05  -0.28  -0.51 | 0.00  -0.22  -0.45  -0.68 | -2.59  -2.77  -2.96  -3.14 | 0.00  -0.18  -0.37  -0.55 |
| Older Siblings (Boys and Girls) | | | | | | | | | | |
| 0  1  2  3  4  5  6  7  8 | 83.08  82.74  82.39  82.05  81.71  81.37  80.35  80.68  80.34 | 0.86  0.73  0.60  0.48  0.35  0.22  0.09  -0.05  -0.17 | 0.00  -0.13  -0.26  -0.39  -0.51  -0.65  -0.77  -0.91  -1.03 | -2.21  -2.34  -2.44  -2.53  -2.62  -2.72  -2.81  -2.91  -3.00 | 0.00  -0.13  -0.23  -0.32  -0.41  -0.51  -0.60  -0.70  -0.79 | 100.25  99.68  99.10  98.52  97.94  97.36  96.79  96.21  95.63 | 0.68  0.54  0.39  0.24  0.09  -0.07  -0.22  -0.38  -0.53 | 0.00  -0.14  -0.29  -0.45  -0.60  -0.75  -0.90  -1.06  -1.21 | -2.15  -2.28  -2.41  -2.54  -2.67  -2.81  -2.94  -3.07  -3.20 | 0.00  -0.13  -0.26  -0.39  -0.52  -0.66  -0.79  -0.92  -1.05 |

**Table S2b. Predicted estimates from best-fit models for weight (kg).** Includes population-specific Z-scores, WHO Z-scores, and Z-score changes at age 2.5 and age 5.0 for each predictor variable (see text and Text S1 for explanation of calculation).

|  | WT  2.5 | Maya WAZ  2.5 | Maya WAZ ∆  2.5 | WHO  WAZ  2.5 | WHO WAZ ∆  2.5 | WT  5.0 | Maya WAZ  5.0 | Maya WAZ ∆  5.0 | WHO  WAZ  5.0 | WHO WAZ ∆  5.0 |
| --- | --- | --- | --- | --- | --- | --- | --- | --- | --- | --- |
| Family Size (Boys and Girls) | | | | | | | | | | |
| 1  2  3  4  5  6  7  8  9 | 11.96  11.82  11.69  11.55  11.41  11.27  11.13  11.00  10.86 | 0.54  0.43  0.32  0.20  0.08  -0.04  -0.16  -0.28  -0.40 | 0.00  -0.11  -0.23  -0.34  -0.46  -0.58  -0.70  -0.82  -0.95 | -0.67  -0.77  -0.86  -0.96  -1.06  -1.16  -1.26  -1.36  -1.46 | 0.00  -0.10  -0.19  -0.28  -0.38  -0.48  -0.59  -0.68  -0.79 | 16.95  16.63  16.31  15.99  15.67  15.35  15.03  14.72  14.40 | 0.79  0.61  0.42  0.23  0.03  -0.17  -0.37  -0.58  -0.80 | 0.00  -0.19  -0.37  -0.57  -0.76  -0.96  -1.17  -1.38  -1.59 | -0.52  -0.66  -0.80  -0.94  -1.08  -1.23  -1.38  -1.53  -1.69 | 0.00  -0.13  -0.27  -0.41  -0.56  -0.71  -0.86  -1.01  -1.17 |
| Younger Siblings (Boys and Girls) | | | | | | | | | | |
| 0  1  2  3 | 12.13  12.19  12.25  12.31 | 0.67  0.72  0.77  0.82 | 0.00  0.05  0.1  0.15 | -0.56  -0.52  -0.48  -0.44 | 0.00  0.04  0.08  0.12 | 16.64  16.14  15.63  15.12 | 0.67  0.34  0.01  -0.34 | 0.00  -0.33  -0.66  -1.01 | -0.65  -0.87  -1.10  -1.34 | 0.00  -0.22  -0.45  -0.69 |
| Older Sibs (Boys and Girls) | | | | | | | | | | |
| 0  1  2  3  4  5  6  7  8 | 12.07  12.10  12.13  12.16  12.19  12.22  12.25  12.28  12.30 | 0.63  0.65  0.68  0.70  0.72  0.75  0.77  0.79  0.81 | 0.00  0.02  0.05  0.07  0.09  0.12  0.14  0.16  0.18 | -0.60  -0.58  -0.56  -0.54  -0.52  -0.50  -0.48  -0.46  -0.44 | 0.00  0.02  0.04  0.06  0.08  0.09  0.11  0.13  0.16 | 16.34  16.58  16.43  16.28  16.13  15.99  15.84  15.69  15.16 | 0.72  0.63  0.53  0.44  0.34  0.25  0.15  0.05  -0.05 | 0.00  -0.09  -0.19  -0.28  -0.38  -0.47  -0.57  -0.67  -0.77 | -0.62  -0.68  -0.74  -0.81  -0.87  -0.94  -1.01  -1.07  -1.13 | 0.00  -0.07  -0.13  -0.20  -0.26  -0.32  -0.39  -0.46  -0.52 |
