# Supplementary material for: Sibling Competition & Growth Tradeoffs. Biological vs. Statistical Significance
Source: PLoS One. 2016 Mar 3;11(3):e0150126. doi: 10.1371/journal.pone.0150126 (PMC4777386; doi:10.1371/journal.pone.0150126)
Supplement: S1 Text — (DOCX) [file pone.0150126.s005.docx]

**Supporting Information**

**Sibling competition & growth tradeoffs. Biological vs. statistical significance**

Karen L Kramer, Amanda Veile and Erik Otárola-Castillo

**S1 Text. Constructing WHO and population-level Z-Scores**

**WHO Z-scores**

The WHO Global Database on Child Growth and Malnutrition (<http://www.who.int/nutgrowthdb/about/introduction/en/index5.html>) uses a Z-score cut-off point of <-2 SD from the median of the WHO standard to classify low height-for-age (stunting) and low weight-for-age (wasting), and to indicate moderate to severe malnutrition. <-3 SD is used as the cut-off to indicate severe undernutrition. The WHO definitions of stunting and wasting are intended to designate a threshold at which growth impairment becomes biologically significant. Indeed stunting and wasting are associated with increased childhood infection and mortality worldwide. However we question the extent to which these cutoffs are relevant for our study population (see below).

World Health Organization (WHO 2006 Z-scores were calculated for the Maya using WHO AnthroPlus software [[76](#_ENREF_76), [77](#_ENREF_77)]. Averages across the sample of 75 children aged 2.5 to 5, height has a mean Z-score of -2.95 for boys and -2.72 for girls, and weight has a mean Z-score of -1.for boys and -1.15 in girls (S1 Fig). Further, 85% (89% of girls; 81% of boys) of children 2.5-5 year old were less than <-2 SD below the median in height and 11.5% (10% of girls, 13% of boys) less than <-2 SD below the median in weight.

We do not, however, find this to be a meaningful reflection of Maya children’s health status. Male adult stature averages 156.1 cm (*SD*=5.1, *n*=47 ages 20-40) for men, and female adult stature 143.2 cm (*SD*=4.3, *n*=58 for ages 20-40). While adult stature is short in global comparison, this is not due to nutritional or epidemiological stress. This is reflected in weight-for-age (S2 Fig) and weight-for-height (not reported here). Children and adults are well nourished and child mortality and morbidity are low [[37](#_ENREF_37)]. The Maya also have high fertility (surviving fertility for women 40 and older is 6.4, *SD* 2.8, *n*=60), suggesting that stature, while it may increase with different dietary inputs and life styles [[56](#_ENREF_56)], does not have long term fitness consequences in this population (98% of children born survive to age 15). Maya stature appears to be mediated by genetic, ecological and macronutrient aspects of their predominantly maize diet, rather than by caloric deficiency. Consequently we construct population-specific Z-scores in conjunction with WHO Z-scores for an additional and more appropriate measure of Maya children’s growth relative to family size.

**Maya population-specific Z-scores**

As part of a previous project, population-specific height and weight Z-scores were calculated for all Maya children ages 0-5. Because infants were included in the original calculation of Z-scores, the growth curves were non-linear and therefore were log-normalized. We continue to use that set of Z-scores in all of our growth papers to maintain consistency. Z-scores were calculated using the following method. The sample was first divided by gender. We then proceeded to create a standard deviate as described in [[78:93-104](#_ENREF_78)]. The natural log of raw height and weight values was computed to normalize the distribution. Age-specific (monthly) height and weight means and standard deviations (z-scores) were then calculated for the logged values for boys and girls using the equation: z=(x-μ)/σ. When population-specific Z-scores are calculated, very few children are less than -2 Z-scores below population mean for height (*n*=5; 7%) or weight (*n*=6; 8%; S2 Fig). The majority are boys, and no values exceed -2.5 Z-scores.
