# Supplementary material for: Sibling Competition & Growth Tradeoffs. Biological vs. Statistical Significance
Source: PLoS One. 2016 Mar 3;11(3):e0150126. doi: 10.1371/journal.pone.0150126 (PMC4777386; doi:10.1371/journal.pone.0150126)
Supplement: S2 Text — (DOCX) [file pone.0150126.s006.docx]

**S2 Text. Fertility, maternal age, height and wealth status**

For the 46 Maya mothers in the sample we examined the relationships of *maternal height* (a proxy for maternal condition) and *wealth* *status* (a proxy for wealth) with mother’s age-specific parity (measured as the number of live births to the mother) using the regression function in R. Maternal age was strongly correlated with parity (*R^2^*=0.53, *p*=0.00). After controlling for maternal age, neither maternal height (*β*=0.04, *p*=0.50) or socioeconomic status (*β*=0.04, *p*=0.75) was a significant predictor of fertility. This may be because fertility is currently in transition, or because of the recent shift to a more cash-dependent economy. Regardless the lack of association suggests that evidence for trade-offs should not be obscured by phenotypic correlations between maternal condition, fertility, and child growth.

**Inclusion of maternal height and wealth status in full and best-fit models**

Although our measures of phenotypic correlation (maternal height and household wealth) were not predictors of age-specific fertility in this cohort of Maya mothers, these variables were retained in two (the family size models) of the four best-fit models (S1 Table). While they were not significant as variables, but were retained because they improved the model’s fit. They may therefore exert subtle, indirect effects on both fertility and child growth through undetected mechanisms.
